# The impact of spliceosome mutations in MDS

**DOI:** 10.1097/HS9.0000000000000218

**Published:** 2019-06-30

**Authors:** Jacqueline Boultwood, Andrea Pellagatti

**Affiliations:** Radcliffe Department of Medicine, University of Oxford, Oxford, United Kingdom

**Take home messages**

- Splicing factor mutations result in different alterations in splicing and largely affect different genes, but these converge in common dysregulated pathways and cellular processes in myelodysplastic syndromes (MDS).
- Splicing factor mutations lead to elevated R-loop formation resulting in an increase in DNA damage in hematopoietic cells.
- The spliceosome may represent a therapeutic vulnerability in patients with myeloid malignancies with splicing factor mutations, and splicing inhibitors are now being evaluated in MDS clinical trials.

## Introduction

Pre-mRNA splicing, a process in which introns are excised from pre-mRNA transcripts to form a mature mRNA, is performed by the spliceosome.^1^,^2^ More than 90% of human protein-coding genes produce multiple mRNA isoforms, contributing to protein diversity.^1^,^2^ It has been long recognized that aberrantly and alternatively spliced mRNA isoforms are often found in cancer and play a role in tumorigenesis. Splicing factor (SF) gene mutations were first identified in patients with myelodysplastic syndromes (MDS) in 2011, as a result of international efforts to sequence the MDS genome(s).^3^,^4^ These mutations occur in >50% of MDS patients and are typically founder mutations, strongly implicating spliceosome dysfunction as a key driver of disease pathophysiology.^1^,^4^,^5^*SF3B1*, *SRSF2*, *U2AF1*, and *ZRSR2* are the most frequently mutated SF genes in MDS,^1^,^4^,^5^ and lead to aberrant 3’ splice site recognition and the generation of aberrantly spliced mRNA transcripts in human and murine bone marrow cells.^1^,^2^,^6^ SF mutations define clinical phenotypes in MDS to some extent, such as the strong association between *SF3B1* mutations and the presence of ring sideroblasts,^3^,^4^ and have differing prognostic impacts.^1^,^2^,^6^*SF3B1* mutations are mutually exclusive with mutations associated with leukemic transformation, in keeping with the good prognosis of *SF3B1*-mutant MDS patients.^1^,^7^ This short review will summarize recent advances in the field.

## Current state of the art

The discovery of SF mutations in MDS has stimulated efforts to investigate their impact on pre-mRNA splicing and hematopoiesis, illuminating how these mutations contribute to the MDS phenotype.

Two recent large studies showed in MDS bone marrow CD34^+^ cells that the common SF mutations resulted in different alterations in splicing and largely affected different genes.^8▪^^,^^9▪^ Alternative exon usage was predominant in *SRSF2*- and *U2AF1*-mutant MDS cases, while *SF3B1* mutations were mainly associated with intron retention events and the use of cryptic 3’ splice sites.^8▪^^,^^9▪^

**Figure 1:**
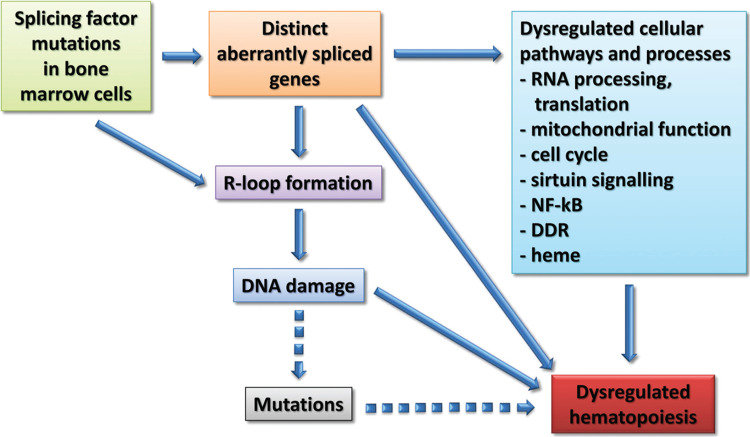
Flowchart showing the different steps through which splicing factor mutations may lead to myelodysplastic syndromes.

The aberrantly spliced genes identified in *SF3B1*, *SRSF2*, and *U2AF1* mutant MDS were shown to converge in common dysregulated cellular processes and pathways, focused on RNA splicing, protein synthesis, and mitochondrial dysfunction, suggesting common mechanisms of action (Fig. 1).^8▪^ Several dysregulated pathways and cellular processes could be linked to MDS pathophysiology, while others, such as sirtuin signaling, represented new players. Importantly, aberrantly splicing events associated with clinical variables were identified, as well as isoforms that independently predicted survival in MDS. Aberrantly spliced genes and dysregulated pathways were also identified in bone marrow erythroid and myeloid precursors of SF-mutant MDS patients.^8▪^ Emerging data from recent studies showed that *SF3B1*, *SRSF2*, and *U2AF1* mutations result in hyperactive NF-κB signaling via aberrant splicing of *MAP3K7*, *CASP8*, and *IRAK4*, respectively.^10▪^^,^^11^

Recent functional studies have illuminated the impact on hematopoiesis of selected aberrantly spliced target genes associated with SF mutations. For example, aberrant splicing of the iron transporter *ABCB7* (in *SF3B1*-mutant cases),^12^*EZH2* (in *SRSF2*-mutant cases),^13^ and *STRAP* and *H2AFY* (in *U2AF1*-mutant cases)^14^ has been linked to some of the hematological abnormalities found in MDS.^2^,^6^,^7^

It is recognized that SRSF2 plays a role in the maintenance of genomic stability.^1^*SRSF2* and *U2AF1* mutations lead to elevated formation of R-loops,^15▪^ structures resulting from the invasion of nascent RNA into DNA. This resulted in increased DNA damage, replication stress, and activation of the ATR-Chk1 pathway.^15▪^ Elevated R-loops also occurred in Srsf2-P95H knock-in mice, and RNase H (which resolves R-loops) partially corrected the growth defect of hematopoietic progenitors in these mice.^15▪^ Moreover, the treatment of U2AF1-S34F expressing cells with ATR inhibitors promoted DNA damage and cell death, with splicing inhibitors enhancing these effects.^16^ Interestingly, aberrant splicing of several genes involved in the suppression/regulation of R-loop formation, including *SETX* and *ATR*, was shown in CD34^+^ cells of SF-mutant MDS patients.^8▪^ R-loop induced DNA damage may give rise to deleterious mutations contributing to the clonal advantage of these cells (Fig. 1).

Conditional knock-in murine models of the common SF mutations (Sf3b1-K700E, Srsf2-P95H, U2af1-S34F) all display some features of MDS, including a macrocytic anemia, with a more pronounced MDS phenotype observed in transplantation experiments using the murine SF-mutant bone marrow cells.^17^–^19^ While a limited overlap was observed between the aberrantly spliced target genes identified in these mice and in SF-mutant MDS/AML patients, the types of aberrant splicing events found were similar.^17^–^19^ Interestingly, several genes implicated in myeloid malignancy, for example, *Stag2* in the Srsf2-P95H model,^18^ showed aberrant splicing as previously reported in patient samples.^7^ It was also shown that the introduction of *Tet2* deletion in the Sf3b1-K700E model^19^ and *Runx1* deletion in the U2af1-S34F model^17^ more accurately mirrored the MDS phenotype, shedding some light on how SF mutations co-operate with other frequently co-occurring mutations.

A common feature of the hematopoietic stem cells (HSCs) from these SF-mutant mouse models is the impaired capacity to reconstitute hematopoiesis in a competitive transplantation setting.^17^–^19^ This observation is surprising given that SF mutations are typically early events in myeloid malignancy and are found in elderly individuals with clonal hematopoiesis of indeterminate potential, suggesting that these mutations confer a growth advantage.^2^ The bone marrow of elderly people may provide a particular environment enabling the expansion of SF-mutant HSCs.

SF mutations are mutually exclusive,^4^,^5^ are not tolerated in a homozygous state,^10▪^ and the survival of SF-mutant cells depends on presence of the wild-type allele.^7^,^13^ This provides the rationale for the targeting of the spliceosome in SF-mutant myeloid malignancy patients. The treatment of SF-mutant patient-derived xenograft models with the spliceosome inhibitors E7107^13^ and H3B-8800^20▪^ has demonstrated the therapeutic potential of these drugs. This has led to rapid clinical interest, with H3B-8800 currently being evaluated in a Phase 1 clinical trial in patients with MDS and other related malignancies. However, some side effects (visual impairment) have been reported in a small proportion of patients with advanced solid tumors treated with E7107 in Phase 1 studies.^7^

## Future perspectives

Our understanding of how SF mutations contribute to the MDS phenotype is growing steadily. Many aberrantly spliced pre-mRNA transcripts have been identified in association with SF mutations, and it is important to confirm the consequences of these splicing variants at the protein level. Further functional studies aiming to determine which of these target genes/pathways play a critical role in disease pathogenesis are also required. How SF mutations cooperate with other frequently co-occurring mutations to give the MDS phenotype is another important and developing area of research.
